# Application of Surface Plasmon Resonance Imaging Biosensors for Determination of Fibronectin, Laminin-5, and Type IV Collagen in Plasma, Urine, and Tissue of Renal Cell Carcinoma

**DOI:** 10.3390/s24196371

**Published:** 2024-09-30

**Authors:** Tomasz Guszcz, Anna Sankiewicz, Lech Gałek, Ewelina Chilinska-Kopko, Adam Hermanowicz, Ewa Gorodkiewicz

**Affiliations:** 1 Department of Urology, Hospital of the Ministry of Interior and Administration in Bialystok, Fabryczna 27, 15-471 Bialystok, Poland; tomasz.guszcz@o2.pl (T.G.); l.galek@zozmswia.bialystok.pl (L.G.); 2 Bioanalysis Laboratory, Faculty of Chemistry, University of Bialystok, Ciolkowskiego 1K, 15-245 Bialystok, Poland; ewka@uwb.edu.pl; 3 Department of Human Anatomy, Medical University of Bialystok, Mickiewicza 2A, 15-230 Bialystok, Poland; ewelina.chilinska-kopko@umb.edu.pl; 4 Department of Pediatric Surgery and Urology, Medical University of Bialystok, Waszyngtona 17, 15-274 Bialystok, Poland

**Keywords:** fibronectin, collagen type IV, laminin-5, renal cell carcinoma, antibody-antigen interaction, biosensor SPRi

## Abstract

Laminin, fibronectin, and collagen IV are pivotal extracellular matrix (ECM) components. The ECM environment governs the fundamental properties of tumors, including proliferation, vascularization, and invasion. Given the critical role of cell-matrix adhesion in malignant tumor progression, we hypothesize that the concentrations of these proteins may be altered in the plasma of patients with clear cell renal cell carcinoma (ccRCC). This study aimed to evaluate the serum, urine, and tissue levels of laminin-5, collagen IV, and fibronectin among a control group and ccRCC patients, with the latter divided into stages T1–T2 and T3–T4 according to the TNM classification. We included 60 patients with histopathologically confirmed ccRCC and 26 patients diagnosed with chronic cystitis or benign prostatic hyperplasia (BPH). Collagen IV, laminin-5, and fibronectin were detected using Surface Plasmon Resonance Imaging biosensors. Significant differences were observed between the control group and ccRCC patients, as well as between the T1–T2 and T3–T4 subgroups. Levels were generally higher in plasma and tissue for fibronectin and collagen IV in ccRCC patients and lower for laminin. The ROC (Receiver operating characteristic) analysis yielded satisfactory results for differentiating between ccRCC patients and controls (AUC 0.84–0.93), with statistical significance for both fibronectin and laminin in plasma and urine. Analysis between the T1–T2 and T3–T4 groups revealed interesting findings for all examined substances in plasma (AUC 0.8–0.95). The results suggest a positive correlation between fibronectin and collagen levels and ccRCC staging, while laminin shows a negative correlation, implying a potential protective role. The relationship between plasma and urine concentrations of these biomarkers may be instrumental for tumor detection and staging, thereby streamlining therapeutic decision-making.

## 1. Introduction

Renal cell carcinoma (RCC) is one of the ten most common cancers in the human population. According to data, in 2023, there were 81,000 new cases of kidney cancer and 15,000 deaths in the United States, 90% of which were RCC [1]. In Europe in 2020, there were 138,611 new cases [2]. Clear cell RCC (ccRCC) accounts for 70–80% of renal cell carcinoma [3].

Metastasis and recurrence rates in ccRCC, as well as its poor prognosis, lead to poor survival rates [4]. The tumor suppressor VHL, an essential component of the cellular oxygen sensor, is lost in 90% of ccRCC cases [5,6]. This leads to a persistent pseudo-hypoxic state, resulting in a robust angiogenic profile of the tumor. Epithelial cells lining the proximal tubule are the cells of origin for ccRCC [7]. They are the most metabolically active cell types in the human body. The transformed epithelial cells are surrounded by a network of blood vessels, interstitial fibroblasts, and inflammatory and immune cells [8]. The cellular tumor components are embedded in the extracellular matrix (ECM), a mix of components with distinct physicochemical and signaling properties. At the molecular level, interactions of tumor cells with the ECM are complex and multifactorial. The ECM environment controls many fundamental properties of tumors, including their proliferation, vascularization, and invasion [9]. Cell–matrix adhesion is an important process for malignant tumor progression. Laminin, fibronectin, and collagen IV are important components of ECM; therefore, we hypothesize that their concentration may be altered in the plasma of ccRCC patients [10]. A review of the literature reveals the potential role of ECM components in ccRCC biology [11], both fibronectin [12,13,14,15], laminin [16,17] and collagen IV [18]. The presence of extracellular matrix (ECM) components, such as collagen IV and fibronectin, plays an integral role in tumor growth, migration, and neovascularization in ccRCC. The expression of COL4A1 (encoding collagen IV chains) and FN1 (encoding fibronectin) is increased in ccRCC tumor samples compared to adjacent tissue [19]. The identification of new prognostic and therapeutic biomarkers has important clinical significance, and we therefore focused on the above-mentioned parameters as possible candidates for that role. The process of metastasis is associated with ECM remodeling, tumor stroma production, and the release of substances into the bloodstream. The researchers have reported higher protein concentrations or activity levels of MMP-2 and MMP-9 in tumor tissue than in normal kidney tissue [20]. The enzymatic effect of the action of metalloproteinases is to disrupt the structure of the extracellular matrix. MMPs enable cell migration by degrading ECM components, including collagens, proteoglycans, and laminins. Excessive ECM degradation can lead to abnormal ECM assembly. The occurrence of more ECM degradation than synthesis can result in the removal of components of the basement membrane (BM) of blood vessels in order to disseminate tumor cells through the circulatory system. This also leads to the unregulated activation of signaling cascades and angiogenesis.

Previous studies have demonstrated the possible significance of laminin, fibronectin, and collagen IV as biomarkers of bladder cancer [21,22] and cutaneous melanoma [23] (**Supplement Materials**). Fibronectin is a multidomain and multifunctional glycoprotein that consists of two disulfide-linked chains. It exists in two biological types, termed plasma and cellular fibronectin. Plasma fibronectin is synthesized by hepatocytes and released into circulation, while cellular fibronectin constitutes the extracellular matrix and is found in all kinds of tissue [24]. Laminin-5, which consists of alpha 2, beta 3, and gamma 2 chains, is a major component of transitional and stratified squamous epithelia, lung mucosa, and other epithelial glands [25]. The collagens are the most abundant proteins in the ECM. Type IV collagen is a major structural protein of basement membranes. It differs chemically and genetically from stromal collagens (types I and III) and cartilage collagen (type II) [26].

The most common methods used for laminin-5, fibronectin, or collagen IV detection are enzyme-linked immunosorbent assay (ELISA) [23], immunohistochemistry [27], or chemiluminescence [28]. However, these techniques are time-consuming and labor-intensive and require labels or the addition of another reagent. Biosensors are devices that are excellent candidates for rapid and sensitive sensing of biomarkers and analysis of biological body changes occurring as a result of disease processes. The surface plasmon resonance (SPR) biosensors are becoming more and more popular. SPR is a non-invasive optical method that can detect even low concentrations of disease biomarkers in various body fluids. One version of the SPR technique is the SPR imaging version (SPRi). SPRi is a technique that integrates the benefits of classic SPR with high-throughput abilities. SPRi measurements can be performed in two systems: a fluidic and a stationary non-fluidic system with multiarray measuring points. SPRi biosensor designs differ according to the types of biomolecules. For example, biorecognition molecules such as antibodies or inhibitors have been successfully used in SPRI biosensors, which have shown promise as potential tools for clinical applications. SPRI has been used for the determination of fibronectin [29], collagen IV [30], laminin-5 [31], and other proteins such as activated leukocyte cell adhesion molecule/CD 166 (ALCAM) and transgelin-2 (TAGLN2) [32], C-reactive protein (CRP) [33], 20S proteasome, and UCH-L1 [34]. All of these components of the extracellular matrix have been determined simultaneously in bladder cancer [21].

This study aimed to assess plasma, urine, and tissue concentrations of laminin-5, collagen IV, and fibronectin in a control group and a group of renal cell carcinoma patients, the latter being divided into non-muscle-invasive and muscle-invasive subgroups. The concentrations of these biomolecules in tumor tissue and tissue from the healthy field of the kidney in the case of partial nephrectomy were also compared.

## 2. Materials and Methods

### 2.1. Reagents

The following reagents were used for the tests: “fibronectin (Sigma, Steinheim, Germany) collagen type IV and laminin 5 as standard solutions for calibration, monoclonal rabbit anti-fibronectin antibody (Sigma, Germany), monoclonal mouse anti-human collagen type IV (Tebubio, Le Perray en Yvelines Cedex France), and rabbit polyclonal antibody specific to human laminin-5 (Abcam, Fremont, CA, USA) as biorecognition element in a biosensor.In addition, was also used: EDC (N-ethyl-N′-(3-dimethylaminopropyl) carbodiimide) (Sigma, Steinheim, Germany), NHS (N-hydroxysuccinimide) (Aldrich, Munich, Germany), carbonate buffer pH = 8.5, 2-aminoethanethiol (cysteamine) (Aldrich, Munich, Germany), human albumin (SIGMA, Steinheim, Germany), absolute ethyl alcohol 99.8% (POCh, Gliwice, Poland), HBS-ES solution (pH = 7.40, 0.01 M HEPES, 0.15 M sodium chloride, 0.005% Tween-20, 3 mM EDTA) and PBS (pH = 7.40, phosphate-buffered saline) (BIOMED, Tokyo, Japan)”. The glass plate with a gold layer constituting the basis of the biosensor came from Ssens (Ssens, Enschede, The Netherlands).

### 2.2. Biological Materials

The samples were obtained from patients with clear cell renal cancer carcinoma who were hospitalized at the J. Sniadecki Provincial Hospital of Bialystok (Poland). The study population was divided into two groups: malignant and control. Plasma, urine, and tissue samples were obtained from patients diagnosed with renal cancer (computer tomography). The cancer diagnosis was confirmed by histological examination of tumor specimens after total or partial nephrectomy. The control group comprises patients with diagnosed benign prostate hyperplasia (diagnosed on USG) or chronic cystitis (persistent inflammation of the bladder diagnosed based on histopathological examination). The control for cancer tissue constituted tissue from the healthy pole of the kidney in case of partial nephrectomy. Patients’ clinical characteristics are presented in Table 1.

Approval (R-I-002/461/2016) for the study was obtained from the Bioethics Committee of the Medical University of Bialystok (Bialystok, Poland), with written informed consent obtained from all patients.

### 2.3. Samples Preparation

Venous blood was collected from the antecubital vein and put into vacuum tubes in a closed system. Venous blood was collected from the antecubital vein and placed into vacuum tubes in a closed system with ethylenediaminetetraacetic acid (EDTA). The samples were centrifuged at 2000× *g* for 10 min at 4 °C. Urine samples were centrifuged at 1850× *g* for 15 min, and the supernatant was separated. Finally, the sample was filtered through a medium-density paper filter. The urine and plasma samples were frozen immediately and maintained at −70 °C. For the determination of concentrations of fibronectin, laminin-5, and collagen IV, immediately before analysis, the prepared plasma and urine samples were diluted with PBS buffer to keep the analyte concentration within the analytically useful range of the calibration curve.

Immediately before analysis, the samples were thawed and diluted to ensure that the analyte concentration remained within the linear range of the calibration curve.

Tissue homogenates were cut into small pieces and divided into 1-g portions. Each portion was homogenized in 0.15 mol L^−1^ KCl using a paddle homogenizer with ice water cooling. The cutting blade’s rotation speed was 10,000 per minute. Next, the homogenate was filtered through nylon fabric with pore sizes of 0.12–0.15 nm. The samples were frozen immediately and placed in a −70 °C freezer.

### 2.4. Determination of Concentration with SPRI Biosensors

#### 2.4.1. Preparation of Biosensors

The biosensor base was a glass chip with a titanium (1 nm) and gold (50 nm) layer, coated with a photopolymer and hydrophobic mask. A layer of Elpemer SD 2047 photopolymer (the main component was novolac epoxy resin) is applied onto the gold chip to isolate twelve active sites with an exposed gold layer at each of the nine sample application locations. A hydrophobic mask (Elpemer SD 2457) is a boundary separating individual groups of measurement points and preventing mixing of analyte solutions.

This meant that there were nine separate places for samples on the chip, with 12 measurement points for each sample (active sites). Next, a linker layer was created on the gold layer. Cysteamine was used as the linker. For this purpose, the chip was immersed in a 20 mM alcoholic solution of cysteamine for at least 12 h; it was next washed with water and ethyl alcohol and then dried in a stream of argon. The next step was to create a layer of the biorecognition element of the biosensor, which was an appropriate antibody. Covalent immobilization was used. The activation of the antibody was performed by addition of EDC (25 µL), NHS (25 µL), and carbonate buffer (10 µL) to 5 µL of antibody. Antibody solutions with the following concentrations were used: fibronectin antibody 5 ng mL^−1^, collagen IV antibody 20 ng mL^−1^, laminin-5 antibody −5 ng mL^−1^. Then, the activated antibody was placed on a thiol (cysteamine)-modified surface and incubated at 37 °C for 1 h. After this time, the active sites of the biosensor were washed with HBS-ES buffer and distilled water. In order to eliminate non-specific adsorption, after incubation of the chip with the antibody, a BSA solution (1 ng mL^−1^) was applied to the active sites of the biosensor and then washed with distilled water. Bovine Serum Albumin (BSA) plays a crucial role in the fabrication of biosensors due to its unique properties. BSA has both hydrophobic and hydrophilic parts on the surface; it can prevent non-specific protein-surface interactions or protein-protein interactions. The BSA prevents the non-specific binding by blocking the leftover spaces over the solid surface after immobilization of a capture biomolecule [35].

**Figure 1:**
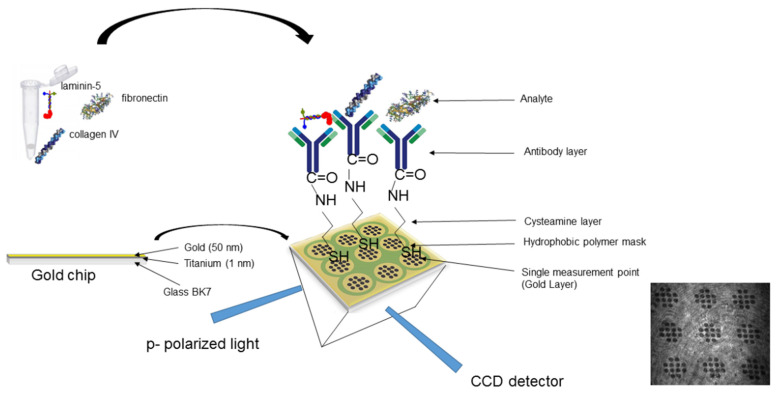
Schematic of the biosensor used.

The biosensor prepared in this way was ready to be used to determine the appropriate analyte (fibronectin, collagen type IV, or laminin-5) in plasma. The schematic of the biosensor used is shown in Figure 1.

#### 2.4.2. SPRI Measurements

SPRi measurements were performed on the apparatus for surface plasmon resonance imaging (SPRi), constructed in the Bioanalysis Laboratory, Faculty of Chemistry, University of Bialystok. The SPRi apparatus comprises a system of polarizers and lenses and a laser He-Ne as the light source. The next element of the SPRI device is the arm with a prism on which the biosensor was placed. The signal is converted by a CCD camera that records the reflected light. The measurement results are presented in the form of a 2D image. The image is analyzed by software (ImageJ NIH, Version 1.8.0_172).

The SPRi technique examines the change in the intensity of reflected monochromatic and p-polarized light after immobilization of subsequent layers constituting the biosensor. P-polarized light has an electric component parallel to the plane of incidence, which couples with the energy of the free electrons (surface plasmons) at the interface and excites their oscillations, which are sensitive to changes on the metal surface. The SPRi signal is proportional to the immobilized mass of the biomolecules on the biosensor. The binding of the free analyte to the immobilized bioreceptor during an SPR experiment causes a change of the refractive index near the surface and, consequently, a change of the resonance angle (SPR). The SPR angle is a characteristic value for a given analytical system because its value is directly dependent on the physicochemical changes occurring at the interface of two media and, therefore, directly proportional to the concentration of the compound. After the analyzed molecule is adsorbed, the intensity of the reflected light changes, and the CCD Camera records that. The image was recorded at a fixed SPR angle twice. The first image was taken after immobilizing the bioreceptor on the biosensor surface, and the second after interaction with the analyte. The signal SPRi was obtained based on contrast values for all the pixels across a particular sample spot that were integrated. Then, the SPRi signal was integrated over the spot area. The difference in the signals obtained from both images constituted the final SPRi signal.

**Figure 2:**
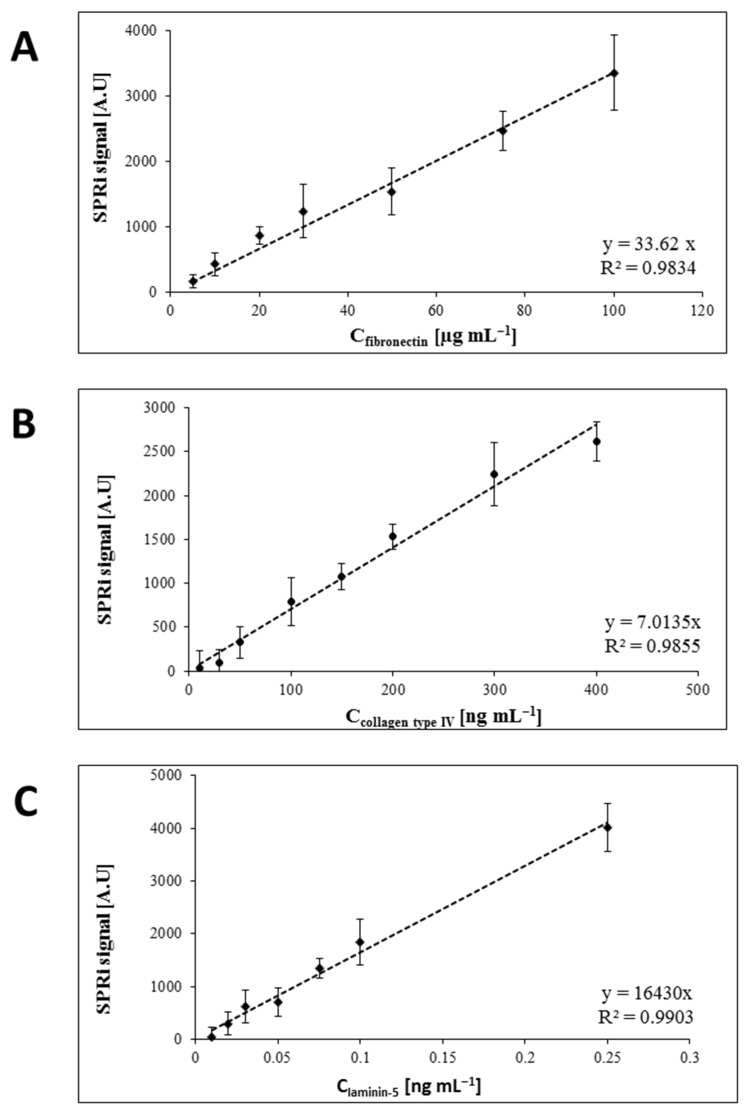
Calibration curve for determination of (**A**) fibronectin, (**B**) collagen type IV and (**C**) laminin-5.

The prepared biosensor with the antibody layer was placed on the prism of the SPRi device. The appropriate angle was selected, and the first photograph was taken. After the mathematical processing of the photos, the SPRI signal for the recognition layer was obtained. Then, 3 µL of plasma, urine, or tissue samples diluted with PBS were applied and left for 10 min. After washing with distilled water and HBS-ES buffer (in order to remove unbound molecules from the surface), the image for the analyte layer was taken at a fixed angle. The final SPRi signal was calculated based on the difference of signals obtained after analysis of the images before and after the interaction of the analyte with the recognition element of the biosensor. Concentrations for the fibronectin, collagen type IV, and laminin-5 were determined on the basis of calibration curves determined immediately before the measurements, considering the appropriate dilutions. The calibration curves obtained are shown in Figure 2. The samples were diluted with PBS buffer to fall concentration within the linear concentration range. Details of the methods used to determine the above-mentioned proteins were presented in previous articles [29,30,31].

### 2.5. Statistical Analysis

Our analysis began with a Shapiro–Wilk test to assess the normal distribution of the data. If the data deviated from normality, we employed non-parametric alternatives: the Mann–Whitney U test for comparing two independent groups and the Kruskal–Wallis test for comparisons across multiple groups. We considered results statistically significant when the p-value fell below 0.05. To determine the most effective threshold for classification, we constructed receiver operating characteristic (ROC) curves, from which we derived the cut-off points that maximized sensitivity and specificity. These points were instrumental in calculating the predictive values for positive and negative outcomes. The optimal cut-off value is calculated on the basis of justified specificities and, using the value m, a calculation tangent to the ROC curve. The setting angle is determined with respect to two values: costs of wrong decisions and morbidity rate.

Costs we considered incorrect decisions to be equal to 1, and the prevalence rate automatically proposed by the program was not changed and was extremely low, around 0.00001. The statistical analyses were performed using PQStat 1.6.4 Software.

## 3. Results

### 3.1. Changes in Plasma Concentration

**Figure 3:**
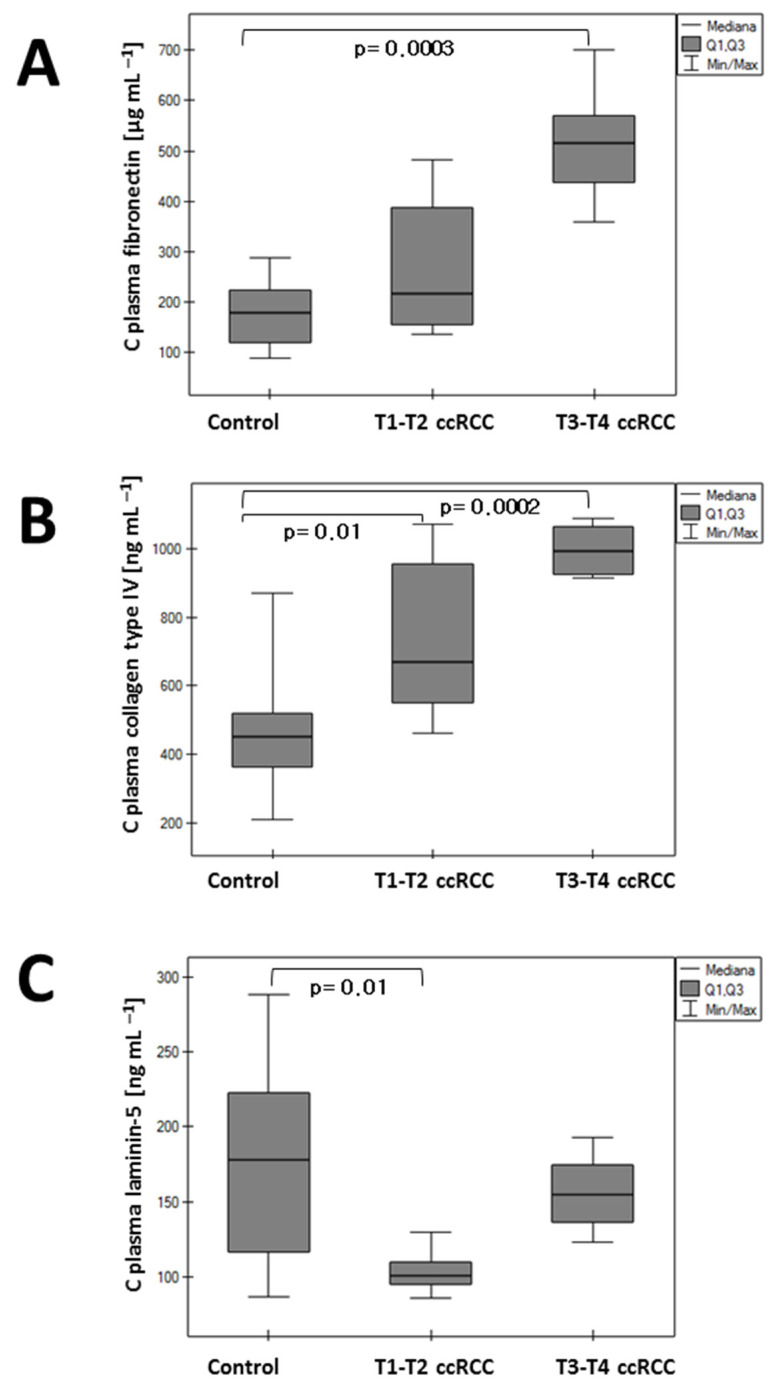
Concentration of fibronectin (**A**), collagen type IV (**B**), and laminin-5 (**C**) in plasma ccRCC and the control group.

There was a significant difference in the plasma concentrations of the analyzed parameters between the control group and renal cancer patients (Figure 3). The patient cohort was divided into subgroups: stages T1–T2, where the cancer is confined to the organ, and stages T3–T4. According to the TNM classification for renal cancer, T1 indicates cancer confined to the organ, T2 is also confined, but with a diameter greater than 7 cm, T3 indicates that the tumor extends beyond the organ without invading the Gerota fascia, and T4 indicates the tumor has invaded the Gerota fascia. The plasma concentration of fibronectin and collagen was found to be elevated in the renal cancer cohort. Notably, there was a statistically significant difference in fibronectin levels between the control group and the T3–T4 group (*p* = 0.0003), as well as for collagen between the control and T1–T2 groups (*p* = 0.01) and between the control and T3–T4 groups (*p* = 0.0002). However, there was no significant difference between the T1–T2 and T3–T4 cohorts. Conversely, plasma laminin concentration was lowered in the T1–T2 cohort (*p* = 0.01). In the T3–T4 cohort, it was slightly lower than in the control group but higher than in the T1–T2 group, although not statistically significant.

### 3.2. Changes in Urine Concentration

**Figure 4:**
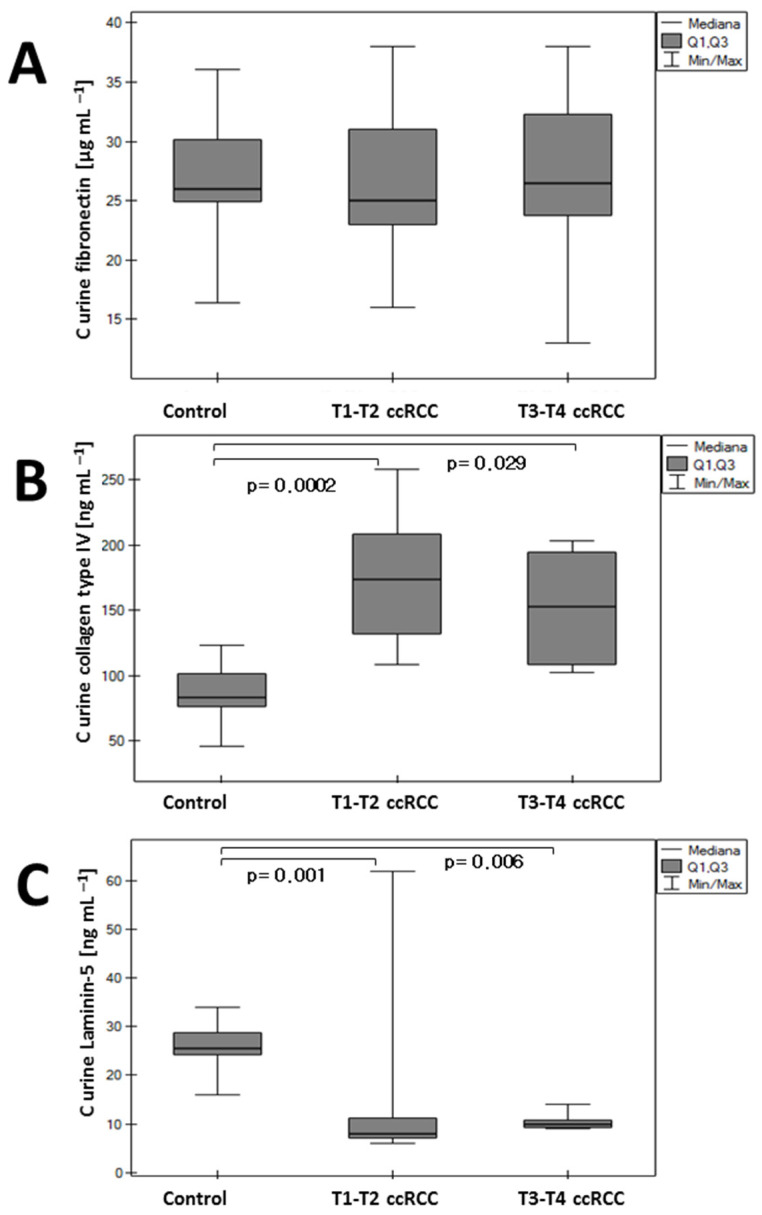
Concentration of (**A**) fibronectin, (**B**) collagen type IV, and (**C**) laminin-5 in urine of ccRCC and the control group.

There were no statistically significant differences in urine concentrations of fibronectin between the control and renal cancer groups (Figure 4). In the case of collagen IV, elevated concentrations were observed for the T1–T2 and T3–T4 cohorts in comparison to the control group, both statistically significant (*p* = 0.0002 and *p* = 0.029, respectively). By contrast, urine laminin concentration was lowered in the cancer cohorts (T1–T2, *p* = 0.001; T3–T4, *p* = 0.006), with no statistically significant differences between T1–T2 and T3–T4.

### 3.3. Changes in Tissue Concentration

**Figure 5:**
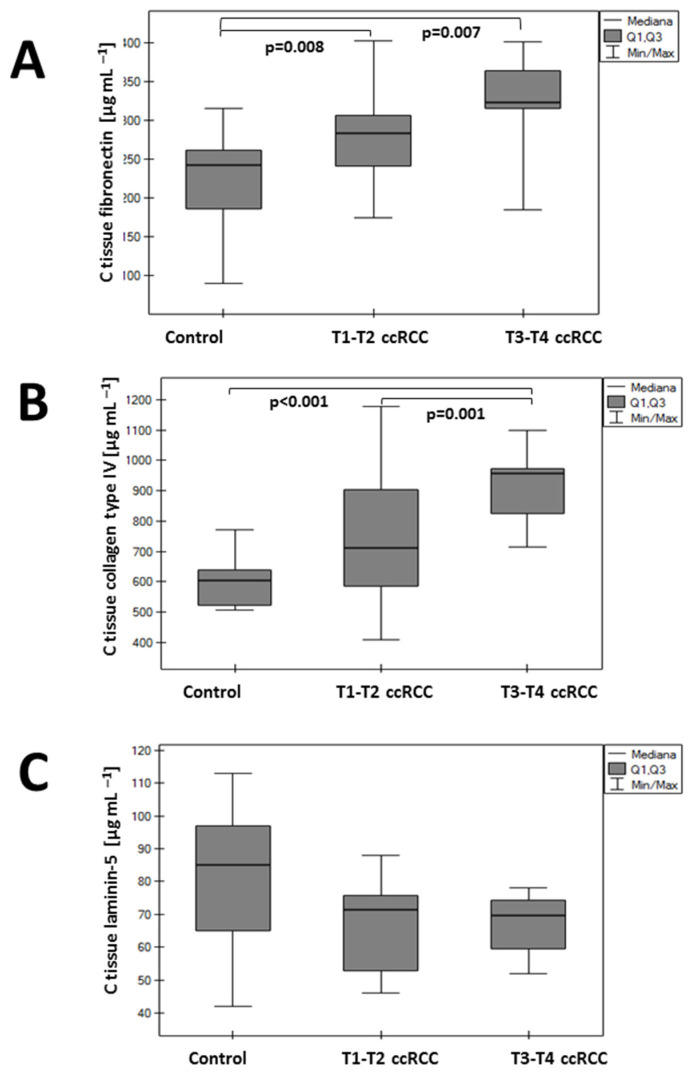
Concentration of fibronectin (**A**), collagen type IV (**B**), and laminin-5 (**C**) in tissue ccRCC and the control group.

In the T1–T2 group, an elevated concentration of fibronectin and type 4 collagen was observed compared with the control group (Figure 5). These values were even higher in the T3–T4 group. Statistical analysis revealed significant differences in fibronectin levels between the control group and the T1–T2 group (*p* = 0.008), control group and the T3–T4 group (*p* = 0.007), as well as for collagen between the control group and the T3–T4 group (*p* < 0.001) and T1–T2 group vs. T3–T4 group (*p* = 0.001). Conversely, tissue laminin concentration was lower in the T1–T2 and T3–T4 cohorts than in the control group, but these differences were not statistically significant.

### 3.4. ROC Analysis

**Figure 6:**
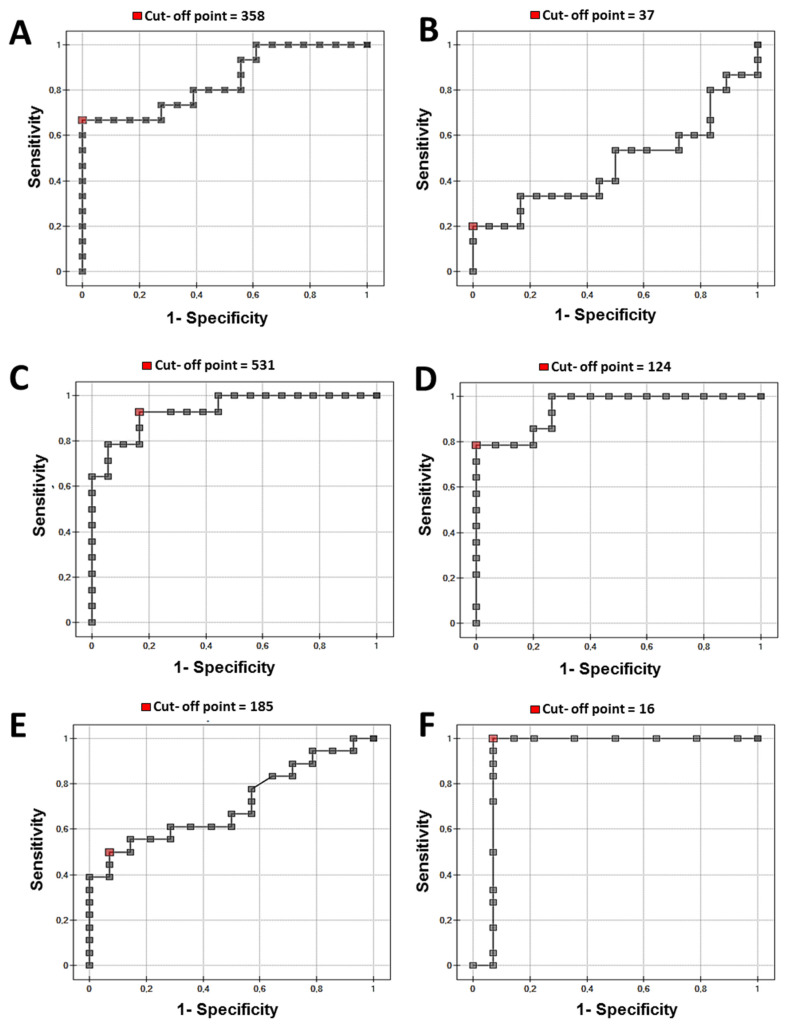
ROC curve (**A**) plasma fibronectin, (**B**) urine fibronectin, (**C**) plasma collagen type IV, (**D**) urine collagen type IV, (**E**) plasma laminin-5, and (**F**) urine laminin-5 ccRCC vs. control (inverted).

**Figure 7:**
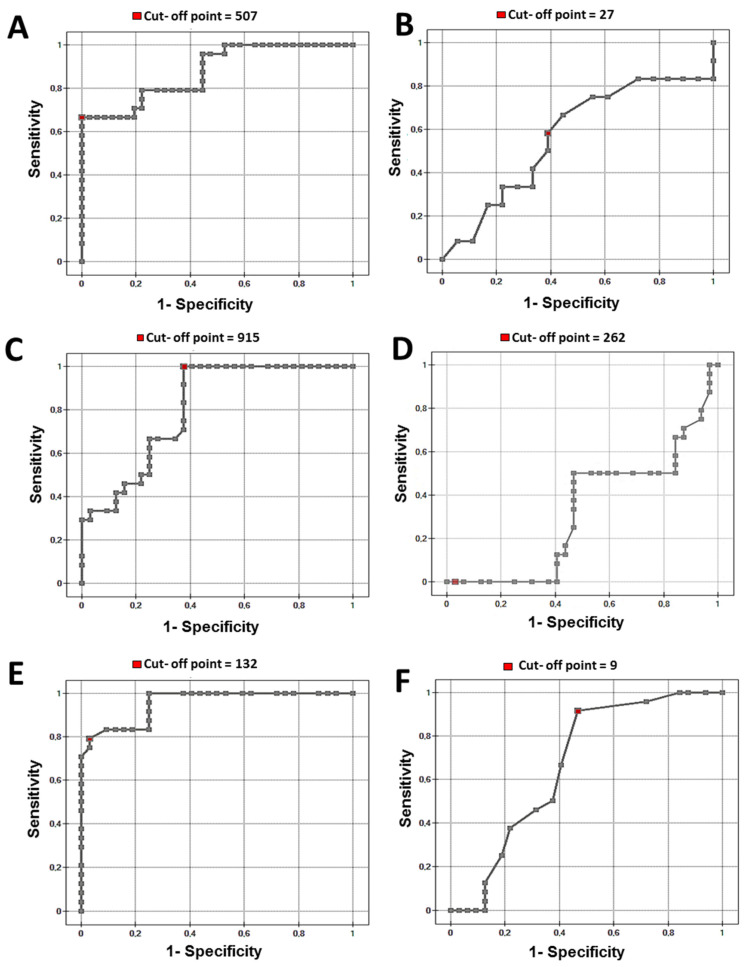
ROC curve (**A**) plasma fibronectin, (**B**) urine fibronectin, (**C**) plasma collagen type IV, (**D**) urine collagen type IV, (**E**) plasma laminin-5, (**F**) urine laminin-5 T1/T2 vs. T3/T4 cRCC.

Analysis of the receiver operating characteristic curves revealed optimal cut-off points of the plasma and urine concentrations of the studied substances for the diagnosis of renal cancer (cut-off points between healthy subjects and renal cancer patients between organ-confined and non-confined groups). For the ROC for fibronectin see Figure 6A,B and Figure 7A,B; for collagen see Figure 6C,D and Figure 7C,D; for laminin-5 see Figure 6E,F and Figure 7E,F. Cut-off points were marked with red dots.

The efficiency of plasma fibronectin and plasma and urine laminin concentration was quite satisfactory for differentiation between the control group and renal cancer (AUC 0.84–0.94) (Table 2). Plasma concentrations of all studied substances were effective for differentiation between the T1–T2 and T3–T4 stages of ccRCC.

## 4. Discussion

In our study, we investigated the concentration of extracellular matrix compounds, explicitly evaluating the levels of fibronectin, laminin, and collagen IV in plasma, urine, and renal cancer tissue (Table 3).

To our knowledge, no such simultaneous examination of these substances has previously been conducted. Utilizing Surface Plasmon Resonance Imaging (SPRi) technology, we could ascertain the levels of these biomolecules, which had been investigated in only a handful of prior studies. Notably, a recent study in 2023 by Guszcz [21] explored these compounds in the context of bladder cancer, reporting that plasma fibronectin concentrations ranged from 176 to 627 µg mL^−1^, with a median of 473 µg mL^−1^. Our renal cancer study’s range was 140 to 715 µg mL-1, with a median of 394 µg mL^−1^. Fibronectin, collagen, and laminin-5 concentrations were determined mainly by ELISA. The concentrations obtained by other researchers were in similar ranges (Fibronectin in serum in patients with epilepsy: 236.96 ± 65.7 ug/mL [36], in chronic hepatitis B (CHB) 413.26 µg/mL [37], collagen IV in patients with breast cancer: 166 ng/mL [38], and laminin-5 in serum in the lung cancer: 0.12-310 ng/mL [39]). In tissues, mainly expression was studied.

Our results were consistent with those of Yocomizo [40], where fibronectin plasma and tissue concentrations were elevated in the T1–T2 group and even more so in the T3–T4 cohort, and with those of Hegele [41], where both localized and metastatic ccRCC groups showed significantly higher levels of plasma concentration compared with the controls.

Bogusławska [42] found that fibronectin gene expression increased five-fold, correlating with tumor grade and poor prognosis, which agrees with the findings of Waalkes [14]. Additionally, Xie J. [13] reported an increase in fibronectin fibril deposition in RCC cells under hypoxic conditions. Brenner [10] showed that invasion of renal cancer is differentially regulated by extracellular matrix compounds, where fibronectin appears to be the most critical factor. Previous suggestions by Yocomizo [40] highlighted fibronectin in tissue and plasma as potential biomarkers for renal cancer. Our data further support the notion that plasma fibronectin may serve as a valuable biomarker for ccRCC, as corroborated by our ROC results.

Overexpression of laminin has been implicated in the advancement of various cancers and is often associated with a poor prognosis. This is evident in cancers such as endometrial carcinoma [43], lung cancer [44], and meningioma [45]. Laminin, primarily located within the basement membrane, plays an important role in many biological processes, including cell adhesion, invasion, and migration. It also participates in signaling pathways that govern cell proliferation and migration. Our research indicates that laminin levels are reduced in advanced renal cancer stages T3–T4 compared with the earlier stages T1–T2. Notably, the concentration in plasma was marginally higher in the T1–T2 group than in the T3–T4 group. For the latter, both plasma and tissue levels of laminin were significantly diminished. These results indicate a potential correlation between elevated laminin levels and improved renal cancer outcomes. However, literature on the prognostic significance of laminin expression in renal cell carcinoma remains scarce. Despite this, some studies, including our own, have indicated that higher laminin expression levels are associated with a more favorable prognosis [17]. Conversely, other studies have identified a negative correlation between laminin concentration and tumor grades, suggesting plasma laminin levels may be a marker for ccRCC diagnosis [16]. The role of collagen IV in ccRCC has yet to be thoroughly examined. Our preliminary findings indicate a positive correlation with fibronectin in both plasma and tissue concentrations, and these parameters are associated with the RCC tumor stage. However, neither fibronectin nor laminin appears to be a reliable predictor for RCC when considered independently. The use of the SPRI biosensor to simultaneously determine these potential biomarkers allows for the expansion of research on their role in the diagnosis of ccRCC.

## 5. Conclusions

In this paper, we concentrated on the extracellular matrix components in renal cell carcinoma patients, namely laminin-5, type IV collagen, and fibronectin. We wanted to check whether the set of potential biomarkers we have studied for bladder cancer [21] will also be used in screening tests for kidney cancer and their differentiation. The problem addressed in the work is significant because, at the moment, doctors have practically no recognized and accepted biomarkers for the diagnosis of kidney cancer. We have extended the study’s simultaneous determination of these biomolecules in different biological materials, i.e., plasma, urine, and tissue. Our pioneering examination of fibronectin, laminin, and collagen IV in plasma, urine, and renal cancer tissue has revealed previously undocumented biomolecule levels, expanding the current understanding of the extracellular matrix in cancer biology. The study showed that fibronectin concentration is increased in the plasma and tissue of patients with ccRCC and may be helpful in staging (T1–T2 vs. T3–T4). Roc analysis showed the potential use of plasma fibronectin concentrations in diagnosing and staging kidney cancer. This is consistent with prior research and indicates its potential role as a biomarker. The collagen concentration in both plasma, tissue, and urine is higher in kidney cancer, but urine shows no difference between the advancement groups. Unlike fibronectin and collagen, laminin shows lower concentrations in kidney cancer patients’ plasma, urine, and tissue. However, only statistically significant differences occur between the control group and the T1-T2 stage in serum and urine. Hence, laminin expression levels tend to decrease in advanced renal cancer stages; our findings indicate that higher levels may be associated with a better prognosis. Our initial findings on the role of collagen IV in ccRCC demonstrate a positive correlation with fibronectin, yet neither fibronectin nor laminin alone can be considered a reliable predictor for ccRCC when assessed independently. The study underscores the need for further research into the role of the extracellular matrix, particularly collagen IV in ccRCC, to validate these compounds’ predictive value and utility in clinical settings.

## Figures and Tables

**Table 1:** Clinical characteristics of patients.

Group	Control Group	BPH (Benign Prostate Hyperplasia)	Cystitis Chronica	ccRCC	TNM T1-T2	TNM T3-T4
Number of patients	26	11	15	60	33	27
Average age	67 (36–86)	71 (55–84)	64 (36–86)	63 (38–83)	62 (38–76)	67 (55–82)
Sex	F (13) M (13)	F (0) M (11)	F (10) M (5)	F (27) M (33)	F (12) M (27)	F (12) M (15)

**Table 2:** Diagnostic efficiency of laminin-5, fibronectin, and collagen IV.

Figure	AUC	*p*-Value	Sensitivity	Specificity	PPV	NPV	Cut-Off
Fibronectin plasma Cancer Presence (6A)	0.84	0.001	66	100	100	78	358
Collagen plasma Cancer Presence (6C)	0.93	0.002	92	83	81	93	531
Laminin plasma Cancer Presence (6E)	0.70	0.040	50	92	90	59	185
Fibronectin urine Cancer Presence (6B)	0.47	0.80	20	100	100	20	37
Collagen urine Cancer Presence (6D)	0.94	0.004	78	100	100	83	124
Laminin urine Cancer Presence (6F)	0.92	0.001	100	92	94	100	16
Fibronectin plasma Cancer Staging (7A)	0.87	0.001	66	100	100	81	507
Collagen plasma Cancer Staging (7C)	0.80	0.001	100	62	65	100	915
Laminin plasma Cancer Staging (7E)	0.95	0.001	79	96	95	86	132
Fibronectin urine Cancer Staging (7B)	0.56	0.380	58	61	50	68	27
Collagen urine Cancer Staging (7D)	0.32	0.020	0	96	0	56	262
Laminin urine Cancer Staging (7E)	0.42	0.030	91	53	59	89	9

**Table 3:** Protein concentrations obtained in the studied groups.

Parameter		Control Median (Range)	T1-T2 N0M0 (TNM) Median (Range)	T3-T4N0M0 (TNM) Median (Range)
Fibronectin [µg mL^−1^]	Plasma	178 (87–288)	222 (140–492)	524 (221–715)
	Urine	26 (16–36)	25 (16–37)	26 (13–38)
	Tissue	242 (90–315)	283 (175–325)	323 (184–401)
Collagen IV [ng mL^−1^]	Plasma	452 (209–869)	668 (461–1071)	994 (915–1088)
	Urine	83 (45–123)	173 (108–258)	153 (104–203)
	Tissue	605 (510–688)	713 (409–1178)	956 (716–1100)
Laminin [ng mL^−1^]	Plasma	178 (87–272)	101 (86–130)	154 (123–193)
	Urine	25 (6–62)	8 (6–62)	10 (9–14)
	Tissue	85 (42–113)	71 (49–88)	69 (52–78)

## Data Availability

Data will be made available on request.

## Supplementary Materials

Table S1. Comparison of proposed biosensors with previously reported sensors [46,47,48,49,50]. https://www.mdpi.com/article/10.3390/s24196371/s1.

## References

[1] Choueiri K.T., Pal S.K., Lewis B., Poteat S., Pels K., Hammers H. (2024). The 5th Kidney Cancer Research Summit: Research Accelerating Cures for Renal Cell Carcinoma in 2023. Oncologist.

[2] Bukavina L., Bensalah K., Bray F., Carlo M., Challacombe B., Karam J.A., Kassouf W., Mitchell T., Montironi R., O’Brien T. (2022). Epidemiology of Renal Cell Carcinoma: 2022 Update. Eur. Urol..

[3] Haake S.M., Rathmell W.K. (2017). Renal cancer subtypes: Should we be lumping or splitting for therapeutic decision making?. Cancer.

[4] Novara G., Ficarra V., Antonelli A., Artibani W., Bertini R., Carini M., Cosciani Cunico S., Imbimbo C., Longo N., Martignoni G. (2010). Validation of the 2009 TNM version in a large multi-institutional cohort of patients treated for renal cell carcinoma: Are further improvements needed?. Eur. Urol..

[5] Nickerson M.L., Jaeger E., Shi Y., Durocher J.A., Mahurkar S., Zaridze D., Matveev V., Janout V., Kollarova H., Bencko V. (2008). Improved identification of von Hippel-Lindau gene alterations in clear cell renal tumors. Clin. Cancer Res..

[6] Iliopoulos O., Levy A.P., Jiang C., Kaelin W.G., Goldberg M.A. (1996). Negative regulation of hypoxia-inducible genes by the von Hippel-Lindau protein. Proc. Natl. Acad. Sci. USA.

[7] Lindgren D., Sjölund J., Axelson H. (2018). Tracing Renal Cell Carcinomas back to the Nephron. Trends Cancer.

[8] Delahunt B., Srigley J.R. (2015). The evolving classification of renal cell neoplasia. Semin. Diagn. Pathol..

[9] Paszek M.J., Zahir N., Johnson K.R., Lakins J.N., Rozenberg G.I., Gefen A., Reinhart-King C.A., Margulies S.S., Dembo M., Boettiger D. (2005). Tensional homeostasis and the malignant phenotype. Cancer Cell.

[10] Brenner W., Groß S., Steinbach F., Horn S., Hohenfellner R., Thüroff J.W. (2000). Differential inhibition of renal cancer cell invasion mediated by fibronectin, collagen IV and laminin. Cancer Lett..

[11] Bond K.H., Chiba T., Wynne K.P.H., Vary C.P.H., Sims-Lucas S., Coburn J.M., Oxburgh L. (2021). The Extracellular Matrix Environment of Clear Cell Renal Cell Carcinoma Determines Cancer Associated Fibroblast Growth. Cancers.

[12] Oxburgh L. (2022). The Extracellular Matrix Environment of Clear Cell Renal Cell Carcinoma. Cancers.

[13] Xie J., Sun M., Zhang D., Chen C., Lin S., Zhang G. (2021). Fibronectin enhances tumor metastasis through B7-H3 in clear cell renal cell carcinoma. FEBS Open Bio.

[14] Waalkes S., Atschekzei F., Kramer M.W., Hennenlotter J., Vetter G., Becker J.U., Stenzl A., Merseburger A.S., Schrader A.J., Kuczyk M.A. (2010). Fibronectin 1 mRNA expression correlates with advanced disease in renal cancer. BMC Cancer.

[15] Ou Y.-C., Li J.-R., Wang J.-D., Chang C.-Y., Wu C.-C., Chen W.-Y., Kuan Y.-H., Liao S.-L., Lu H.-C., Chen C.-J. (2019). Fibronectin Promotes Cell Growth and Migration in Human Renal Cell Carcinoma Cells. Int. J. Mol. Sci..

[16] Qian H., Li X., Zhang W., Ma L., Sun J., Tang X., Chen Y., Teng L., Wang W., Li D. (2018). Caspase-10, matrix metalloproteinase-9 and total laminin are correlated with the tumor malignancy of clear cell renal cell carcinoma. Oncol. Lett..

[17] Bai J., Zheng A., Ha Y., Xu X., Yu Y., Lu Y., Zheng S., Shen Z., Luo B., Jie W. (2022). Comprehensive analysis of LAMC1 expression and prognostic value in kidney renal papillary cell carcinoma and clear cell carcinoma. Front. Mol. Biosci..

[18] Soupir A.C., Hayes M.T., Peak T.C., Ospina O., Chakiryan N.H., Berglund A.E., Stewart P.A., Nguyen J., Segura C.M., Francis N.L. (2023). Increased spatial coupling of integrin and collagen IV in the immunoresistant clear cell renal cell carcinoma tumor microenvironment. BioRxiv.

[19] Xia B., Wang J., Zhang D., Hu X. (2024). Integration of basement membrane-related genes in a risk signature for prognosis in clear cell renal cell carcinoma. Sci. Rep..

[20] Qiao Z.K., Li Y.L., Lu H.T., Wang K.L., Xu W.H. (2013). Expression of tissue levels of matrix metalloproteinases and tissue inhibitors of metalloproteinases in renal cell carcinoma. World J. Surg. Oncol..

[21] Guszcz T., Sankiewicz A., Gorodkiewicz E. (2023). Application of surface plasmon resonance imaging biosensors for determination of fibronectin, laminin-5 and type IV collagen in serum of transitional bladder cancer patients. J. Pharm. Biomed. Anal..

[22] Dong F., Shen Y., Xu T., Wang X., Gao F., Zhong S., Chen S., Shen Z. (2018). Effectiveness of urine fibronectin as a non-invasive diagnostic biomarker in bladder cancer patients: A systematic review and meta-analysis. World J. Surg. Oncol..

[23] Tas F., Bilgin E., Karabulut S., Duranyildiz D. (2016). Clinical significance of serum laminin and type-IV collagen levels in cutaneous melanoma patients. Mol. Clin. Oncol..

[24] Wang K., Seo B.R., Fischbach C., Gourdon D. (2016). Fibronectin Mechanobiology Regulates Tumorigenesis. Cell. Mol. Bioeng..

[25] Lohi J. (2001). Laminin-5 in the progression of carcinomas. Int. J. Cancer.

[26] Liotta L.A., Tryggvason K., Garbisa S., Hart I., Foltz C.M., Shafie S. (1980). Metastatic potential correlates with enzymatic degradation of basement membrane collagen. Nature.

[27] Miyake M., Hori S., Morizawa Y., Tatsumi Y., Toritsuka Ohnishi M., Shimada K., Furuya H., Khadka V.S., Deng Y., Ohnishi K. (2017). Collagen type IV alpha 1 (COL4A1) and collagen type XIII alpha 1 (COL13A1) produced in cancer cells promote tumor budding at the invasion front in human urothelial carcinoma of the bladder. Oncotarget.

[28] Nakagawa M., Karashima T., Kamada M., EYoshida, Yoshimura T., Nojima M., Inoue K., Shuin T., Seiki M., Koshikawa N. (2017). Development of a fully automated chemiluminescence immunoassay for urine monomeric laminin-γ2 as a promising diagnostic tool of non-muscle invasive bladder cancer. Biomark. Res..

[29] Sankiewicz A., Romanowicz L., Pyc M., Hermanowicz A., Gorodkiewicz E. (2018). SPR imaging biosensor for the quantitation of fibronectin concentration in blood samples. J. Pharm. Biomed. Anal..

[30] Sankiewicz A., Lukaszewski Z., Trojanowska K., Gorodkiewicz E. (2016). Determination of collagen type IV by Surface Plasmon Resonance Imaging using a specific biosensor. Anal. Biochem..

[31] Sankiewicz A., Romanowicz L., Laudanski P., Zelazowska-Rutkowska B., Puzan B., Cylwik B., Gorodkiewicz E. (2016). SPR imaging biosensor for determination of laminin-5 as a potential cancer marker in biological material. Anal. Bioanal. Chem..

[32] Ladd J., Taylor A.D., Piliarik M., Homola J., Jiang S. (2009). Label-free detection of cancer biomarker candidates using surface plasmon resonance imaging. Anal. Bioanal. Chem..

[33] Vance S., Sandros M. (2014). Zeptomole Detection of C-Reactive Protein in Serum by a Nanoparticle Amplified Surface Plasmon Resonance Imaging Aptasensor. Sci. Rep..

[34] Sankiewicz A., Guszcz T., Gorodkiewicz E. (2021). Application of SPRi Biosensors for Determination of 20S Proteasome and UCH-L1 Levels in the Serum and Urine of Transitional Bladder Cancer Patients. Appl. Sci..

[35] Jeyachandran Y.L., Mielczarski J.A., Mielczarski E., Rai B. (2010). Efficiency of blocking of non-specific interaction of different proteins by BSA adsorbed on hydrophobic and hydrophilic surfaces. J. Colloid Interface Sci..

[36] Xi Z.Q., Wang X., Luo J., Wang W., Xiao F., Chen D., Wang S., Li M., Wang L. (2015). Fibronectin is a potential cerebrospinal fluid and serum epilepsy biomarker. Epilepsy Behav..

[37] Erturk A., Cure E., Ozkurt Z., Parlak E., Cure M.C. (2014). Serum fibronectin levels in acute and chronic viral hepatitis patients. Malays. J. Med. Sci..

[38] Mazouni C., Arun B., André F., Ayers M., Krishnamurthy S., Wang B., Hortobagyi G.N., Buzdar A.U., Pusztai L. (2008). Collagen IV levels are elevated in the serum of patients with primary breast cancer compared to healthy volunteers. Br. J. Cancer.

[39] Tas F., Bilgin E., Tastekin D., Erturk K., Duranyildiz D. (2016). Clinical significance of serum laminin levels in patients with lung cancer. Biomed. Rep..

[40] Yokomizo A., Takakura M., Kanai Y., Sakuma T., Matsubara J., Honda K., Naito K., Yamada S.T., Ono M. (2012). Use of quantitative shotgun proteomics to identify fibronectin 1 as a potential plasma biomarker for clear cell carcinoma of the kidney. Cancer Biomark..

[41] Hegele B., Kosche A.J., Schrader S., Sevinc P.J., Olbert R., Hofmann J., Kropf D. (2008). Transitionalzellkarzinom der Harnblase, Evaluation von zellul¨arem Fibronektin im Plasma als stadienabh¨angiger Marker [Transitional cell carcinoma of the bladder. Evaluation of plasma levels of cellular fibronectin as a stage-dependent marker]. Urologe.

[42] Boguslawska J., Kedzierska H., Poplawski P., Rybicka B., Tanski Z., Piekielko-Witkowska A. (2016). Expression of Genes Involved in Cellular Adhesion and Extracellular Matrix Remodeling Correlates with Poor Survival of Patients with Renal Cancer. J. Urol..

[43] Kunitomi H., Kobayashi Y., Wu R.C., Takeda T., Tominaga E., Banno K., Aoki D. (2020). LAMC1 is a prognostic factor and a potential therapeutic target in endometrial cancer. J. Gynecol. Oncol..

[44] Teng Y., Wang Z., Ma L., Zhang L., Guo Y., Gu M., Wang Z., Wang Y., Yue W. (2016). Prognostic significance of circulating laminin gamma2 for early-stage non-small-cell lung cancer. OncoTargets Ther..

[45] Ke H.L., Ke R.H., Li B., Wang X.H., Wang Y.N., Wang X.Q. (2013). Association between laminin γ1 expression and meningioma grade, recurrence, and progression-free survival. Acta Neurochir..

[46] Prabowo B.A., Sousa C., Cardoso S., Freitas P., Fernandes E. (2022). Labeling on a Chip of Cellular Fibronectin and Matrix Metallopeptidase-9 in Human Serum. Micromachines.

[47] Chen C.-Y., Chang C.-C., Yu C., Lin C.-W. (2012). Clinical Application of Surface Plasmon Resonance-Based Biosensors for Fetal Fibronectin Detection. Sensors.

[48] Singh L., Singh R., Kumar S., Zhang B., Kaushik B.K. (2020). Development of Collagen-IV Sensor Using Optical Fiber-Based Mach-Zehnder Interferometer Structure. IEEE J. Quantum Electron..

[49] Li L., Niu C., Li T., Wan Y., Zhou Y., Wang H., Yuan R., Liao P. (2018). Ultrasensitive electrochemiluminescence biosensor for detection of laminin based on DNA dendrimer-carried luminophore and DNA nanomachine-mediated target recycling amplification. Biosens Bioelectron.

[50] Zhou J., Han T., Ma H., Yan T., Pang X., Li Y., Wei Q. (2015). A novel electrochemiluminescent immunosensor based on the quenching effect of aminated graphene on nitrogen-doped carbon quantum dots. Anal Chim Acta..

