# Supplementary material for: Application of Surface Plasmon Resonance Imaging Biosensors for Determination of Fibronectin, Laminin-5, and Type IV Collagen in Plasma, Urine, and Tissue of Renal Cell Carcinoma
Source: Sensors (Basel). 2024 Sep 30;24(19):6371. doi: 10.3390/s24196371 (PMC11478812; doi:10.3390/s24196371)
Supplement: Supplementary file 1 [file sensors-24-06371-s001.zip › sensors-3201199-supplementary.pdf]

Supplementary File.

Comparison of proposed biosensors with previously reported sensors. Biosensors used in our studies to assess selected ECM components allow for the simultaneous detection of fibronectin, collagen IV, and laminin 5 in various biological materials. Table S1 compares the parameters of the tested SPRi biosensors with sensors previously presented in the literature.

Table S1. Comparison of proposed biosensors with previously reported sensors.

The biomolecule being determined	Detection method	Linear range	Limit detection (LOD)	References
Fibronectin	microfluidic chip	0.2- 10 μg mL^{-1}	205.14 ng mL^{-1}	[1]
	SPR biosensor	0.50 - 100 ng mL^{-1}	no data	[2]
	SPRi biosensor (presented in work)	0.001 – 0.250 ng mL^{-1}	4 ng mL^{-1}	
Collagen IV	LSPR biosensor	2.0 - 40 μg mL^{-1}	1.6 μg mL^{-1}	[3]
	SPRi biosensor (presented in work)	5.00 – 400 ng mL^{-1}	2.4 ng mL^{-1}	
Laminin	electrochemiluminescence biosensor	0.10 pg mL^{-1} -100 ng mL^{-1}	0.0661 pg mL^{-1}	[4]
	electrochemiluminescent immunosensor	10 – 100 ng mL^{-1}	3.2 ng mL^{-1}	[5]
	SPRi biosensor (presented in work)	0.001 – 0.250 ng mL^{-1}	4 pg ng mL^{-1}	

References.

[1] Prabowo, B.A.; Sousa, C.; Cardoso, S.; Freitas, P.; Fernandes, E. Labeling on a Chip of Cellular Fibronectin and Matrix Metallopeptidase-9 in Human Serum. Micromachines 2022, 13, 1722.

[2] Chen, C.-Y.; Chang, C.-C.; Yu, C.; Lin, C.-W. Clinical Application of Surface Plasmon Resonance-Based Biosensors for Fetal Fibronectin Detection. *Sensors* 2012, *12*, 3879-3890.

[3] L. Singh, R. Singh, S. Kumar, B. Zhang and B. K. Kaushik, "Development of Collagen-IV Sensor Using Optical Fiber-Based Mach-Zehnder Interferometer Structure," in IEEE Journal of Quantum Electronics, vol. 56, no. 4, pp. 1-8, Aug. 2020, Art no. 7700208, doi: 10.1109/JQE.2020.3003022.

[4] Li L, Niu C, Li T, Wan Y, Zhou Y, Wang H, Yuan R, Liao P. Ultrasensitive electrochemiluminescence biosensor for detection of laminin based on DNA dendrimer-carried luminophore and DNA nanomachine-mediated target recycling amplification. Biosens Bioelectron. 2018 Mar 15;101:206-212. doi: 10.1016/j.bios.2017.10.009.

[5] Zhou J, Han T, Ma H, Yan T, Pang X, Li Y, Wei Q. A novel electrochemiluminescent immunosensor based on the quenching effect of aminated graphene on nitrogen-doped carbon quantum dots. Anal Chim Acta. 2015 Aug 19;889:82-9. doi: 10.1016/j.aca.2015.07.018
